# Workplace Violence Among Healthcare Workers in a Tertiary Medical City in Riyadh: A Cross-Sectional Study

**DOI:** 10.7759/cureus.14836

**Published:** 2021-05-04

**Authors:** Fares F Alharbi, Nowar A Alzneidi, Ghaida H Aljbli, Sarah A Morad, Ettab G Alsubaie, Mahmoud A Mahmoud, Sami A Al-Dubai, Firas A Nakshabandi, Saleh bin Saleh

**Affiliations:** 1 College of Medicine, King Saud Bin Abdulaziz University for Health Sciences, Riyadh, SAU; 2 Department of Mental Health, Ministry of the National Guard Health Affairs, Riyadh, SAU; 3 Department of Research Office, King Abdullah International Medical Research Center, Riyadh, SAU; 4 Department of Family Medicine, King Khalid University Hospital, Riyadh, SAU; 5 Department of Plastic Surgery, Ministry of the National Guard Health Affairs, Riyadh, SAU; 6 Department of Pediatrics, King Fahad Medical City, Riyadh, SAU; 7 Department of Public Health, Imam Muhammad Ibn Saud Islamic University, Riyadh, SAU; 8 Joint Program of Preventive Medicine Post Graduate Studies, Saudi Ministry of Health, Medina, SAU; 9 Department of Psychiatry and Behavioral Neuroscience, The University of Chicago, Chicago, USA; 10 Mental Health Department, Empathic Resonance, LLC, Chicago, USA; 11 Department of Medicine, Ministry of the National Guard Health Affairs, Riyadh, SAU

**Keywords:** violence, sexual harassment, health personnel, workplace, saudi arabia

## Abstract

Introduction

Workplace violence is a common problem that is encountered by healthcare workers worldwide; however, it is still under-studied in Saudi Arabia. This study aims to determine the prevalence of workplace violence and to explore reasons for not reporting it among healthcare workers in a tertiary medical city in Riyadh.

Methods

This cross-sectional study was conducted among 404 healthcare workers who had direct contact with patients or their relatives in a tertiary care medical city in Riyadh, Saudi Arabia. Data were analyzed using Statistical Analysis Software Package (SPSS; IBM, Armonk, NY, USA).

Results

Most participants (81.4%) had experienced verbal, physical, academic, or sexual violence. Approximately 39.6% of those who experienced workplace violence did not report the incident, and the most common reason for not reporting was identified as “reporting would not accomplish anything” (49.4%). About 27.5% of violence victims did not know how to report the incidents. Patients or their relatives were the main sources of violence across all violence categories except academic violence, in which consultant physicians were the main source.

Conclusions

The prevalence of workplace violence in the population studied was higher than anticipated compared to similar studies both in Saudi Arabia and globally. Almost half of those who were subjected to violence did not report the incident, believing that reporting would not change anything. There is arguably an urgent need to develop strategies that reduce workplace violence and facilitate reporting it in hospitals. Moreover, awareness programs regarding the negative impacts of violence against healthcare workers on the quality of care are necessary.

## Introduction

Violence against healthcare workers is associated with various psychological, physical, and occupational impacts that may affect the quality of the provided care. Anxiety [1], depression [1], post-traumatic stress disorder (PTSD) [2], and burnouts [2] were all reported among healthcare workers as psychological consequences of workplace violence. Physical symptoms were also reported after exposure to violent incidents; these include pain, headache, and stomach ache [3]. Also, up to 65% of healthcare workers sustained physical injuries due to workplace violence, and some of the victims required medical treatment [3]. Lacerations, bites, abrasions, and bruises were all consequences of physical workplace violence [3]. Being subjected to violence also affects the victims occupationally; it is associated with decreased productivity [4], decreased job satisfaction [5], increased absence [6], and the intention to quit the job [7].

Violence against healthcare workers is a global problem [8]. In a recent review, the 12-month prevalence of violence against healthcare providers was found to be 70.9% in Australia and New Zealand, 67.3% in North America, 64.9% in Asia, 62.7% in Latin America, 59.2% in Africa, and 48.1% in Europe [8]. Non-physical violence (42.5%) was more common than physical violence (24.4%), with verbal abuse (57.6%) being the most common type of non-physical violence and sexual harassment (12.4%) the least common [8].

There are a number of studies that examined workplace violence among healthcare workers in Saudi Arabia [9-16]. However, most of these studies were limited to a specific occupation like nursing [9,10], or a specific healthcare setting like emergency departments [11,12] or primary care centers [13,14]. To the best of our knowledge, there are only two Saudi studies that addressed workplace violence among all healthcare workers without being limited to a specific department or healthcare setting [15,16]. The first study was conducted in Arar city by Al Anazi et al., which found that there was a 48.6% prevalence of workplace violence among healthcare workers working in general hospitals and primary care centers [15]. The second study was in Abha City, where the prevalence of workplace violence was found to be 57.5% [16]. Despite the high rates of workplace violence in Saudi Arabia, the prevalence of reporting violent incidents ranged from 1.7% [14] to 38.5% [12].

Due to the aforementioned negative consequences of workplace violence, there is arguably an urgent need to identify the extent of workplace violence among a broader range of healthcare providers across a wider range of settings in Saudi Arabia. Also, identifying reasons that prevent victims from reporting violent incidents can promote the development of workplace policies that facilitate reporting. Thus, the aim of this study is to determine the prevalence of workplace violence amongst all healthcare workers in a tertiary medical city in Riyadh and to identify the reasons behind not reporting incidents of violence. To the best of our knowledge, this is the first study in Riyadh that examines workplace violence among all healthcare workers in a tertiary medical city without being limited to a specific department or occupation.

## Materials and methods

Study participants, settings, and sample size

This cross-sectional study included all health care providers that have direct contact with patients or their relatives in a tertiary medical city in Riyadh, Saudi Arabia. The medical city includes medical and surgical wards, pediatric wards, intensive care units, a cardiac center, an emergency and trauma department, obstetrics and gynecology services, antenatal and postpartum wards, rehabilitation and long-term care wards, outpatient clinics, and dental services. It also includes 11 primary healthcare centers that are distributed around different neighborhoods in Riyadh. Moreover, it is designated as a military medical city, providing care to the Saudi national guard members, their families, and other eligible individuals.

For a population size of 5,447 employees, the optimal sample size to obtain results within a margin of error of 5% and a confidence level of 95% was identified as 359 employees (n=359). However, the questionnaire was given to 450 employees to ensure a better response; 404 questionnaires were returned with a response rate of 89.8%. All participants were aware of the study objectives and signed a consent form before filling out the questionnaire.

Data collection method

A self-administered survey was distributed to healthcare workers in a tertiary medical city in Riyadh, Saudi Arabia from July 1, 2019 to October 1, 2019. The survey was conducted in English because it is the language spoken by all the staff. The survey was adapted from a previously published and validated study [17]. It includes seven sections: 1) demographics, 2) verbal violence, 3) physical violence, 4) academic violence, 5) sexual harassment, 6) reasons for not reporting violence, and 7) the influence of violence on the participants. The definition of verbal violence included shouting, humiliating, or speaking to the person in an un-respectful manner. Physical violence was defined as any threat that could cause physical injuries like hitting, pushing, or slapping. The definition of academic violence was being pressured to do personal services that are not related to the duties of the employee. Sexual harassment was defined as being subjected to repeated staring, comments or jokes against gender or body figure, or inappropriate touching of a sexual nature. It also includes being offered unwanted gifts with sexual underpinnings [17]. This study was IRB-approved by King Abdullah International Medical Research Center (IRB no. RYD-18-419812-203457). Informed consent was obtained from the participants.

Data management and analysis

Data were analyzed by using the SPSS (Statistical Analysis Software Package) program version 22 (IBM, Armonk, NY, USA). Descriptive analysis was employed to obtain the mean and standard deviation (SD) for the continuous variables and to obtain the frequency and percentage for the categorical variables. P-value was considered statistically significant if P < 0.05.

## Results

Socio-demographic characteristics of the participants

In this study, the age range of the participants was 24-69 years old, with a mean of 36.9 years old and an SD of 8.5 years. Most participants were >35 years old (52.5%), females (65.1%), Saudis (54.0%), and nurses (39.1%). Most of them had an experience of six years or more (78.2%) and have been working at the current hospital for six years or more (60.1%) (Table 1).

**Table 1:** Sociodemographic characteristics of the participants (n=404).

Variables	No. (%)
Age	
≤35	192 (47.5)
>35	212 (52.5)
Gender	
Male	141 (34.9)
Female	263 (65.1)
Nationality	
Saudi	218 (54.0)
Arab	38 (9.4)
Asian	148 (36.6)
Job title	
Physician	137 (33.9)
Nurse	158 (39.1)
Administrator	49 (12.1)
Technician	37 (9.2)
Other	23 (5.7)
Specialty	
Surgery	6 (1.5)
Medicine	123 (30.4)
Pediatrics	76 (18.8)
Obstetrics and gynecology	36 (8.9)
Emergency	23 (5.7)
Intensive care	22 (5.4)
Psychiatry	38 (9.4)
Other	79 (19.6)
Years of experience	
1 to 2 years	38 (9.4)
3 to 5 years	50 (12.4)
6 years or more	316 (78.2)
How many years you have been working at this hospital?	
1 to 2 years	67 (16.6)
3 to 5 years	94 (23.3)
6 years or more	243 (60.1)

Verbal violence among participants

Verbal violence was reported by 79.5% of the participants. The most common type of verbal abuse was “Someone was Inappropriately nasty, rude, or verbally hostile to them” (69.1%) and “Someone spoke to them un-respectfully” (67.8%). The main sources of verbal violence were patients or their relatives (60.1%) and consultants (45.5%). Verbal violence has happened more frequently in the emergency (40.3%) and obstetrics and gynecology departments (32.9%). The majority had witnessed an episode of verbal violence, and they reported that both males and females were frequently verbally harassed (56.7%). The majority (42.3%) of the participants reported that their experiences with verbal violence were very upsetting and of major importance (Table 2).

**Table 2:** Verbal violence among participants (n=404).

Variables	No. (%)
Experienced verbal violence	321 (79.5)
Did you experience any of the following types of “verbal violence”?	
Someone shouted at you	257 (63.6)
Someone was Inappropriately nasty, rude, or verbally hostile to you	279 (69.1)
Someone belittled or humiliated you during meetings or rounds	178 (44.1)
Someone was joking at you	148 (36.6)
Someone spoke to you un-respectfully	274 (67.8)
Others	30 (7.4)
None of the above	83 (20.5)
In which department/s these types of verbal violence have happened more frequently?	
Surgery	78 (20.5)
Medicine	73 (19.2)
Pediatrics	40 (10.5)
Ob\gyn	125 (32.9)
Emergency	153 (40.3)
ICU	51 (13.4)
Psychiatry	14 (3.7)
Other	80 (21.1)
Who was/were the main source/s of verbal violence?	
Consultant	184 (45.5)
Specialist/registrars	10 (2.5)
Residents	116 (28.7)
Nurses	61 (15.1)
Patients or their relatives	243 (60.1)
During your career, have you witnessed any episodes of verbal violence?	
Yes	357 (88.4)
No	47 (11.6)
From your experience, who is, in general, most frequently verbally harassed?	
Male	50 (12.4)
Female	125 (30.9)
Both	229 (56.7)
If you experience any form of verbal violence, do you describe this/these experience/s as being very upsetting and of major importance?	
Yes, all of them	171 (42.3)
Yes, some of them	196 (48.5)
No, not all	36 (8.9)

Physical violence among participants

Physical violence was reported by 67.6% of the participants. About one-third of the participants had been threatened with physical harm (33.4%), and 26.0 % had been subjected to a slap, push, hit, kick, or having things thrown at them. About 25.7% had been placed at unnecessary medical risk. Physical violence had occurred more frequently in the emergency (18.1%) and surgery departments (16.6%). The main sources of physical violence were patients or their relatives (32.9%) and consultants (13.4%). During their career, 80.7% of the participants had witnessed an episode of physical violence, and they reported that both males and females (54.9%) were frequently physically harassed. The majority (50.7%) of the participants reported that their experiences with physical violence were very upsetting and of major importance (Table 3).

**Table 3:** Physical violence among participants (n=404).

Variables	No. (%)
Experienced physical violence	273 (67.6)
Did you experience any of the following types of physical violence?	
Someone threatened you with physical harm	135 (33.4)
slap, push, hit, kick or having things thrown at you	105 (26.0)
Someone placed you at unnecessary medical risks	104 (25.7)
Others	95 (23.5)
non-of the above	131 (32.4)
in which department/s these types of physical violence have happened more frequently?	
Surgery	67 (16.6)
Medicine	58 (14.4)
Pediatrics	6 (1.5)
Ob\gyn	26 (6.4)
Emergency	73 (18.1)
ICU	18 (4.5)
Psychiatry	5 (1.2)
Other	33 (8.2)
Who was/were the main source/s of physical violence?	
Consultant	54 (13.4)
Specialist/registrars	1 (0.2)
Residents	25 (6.2)
Nurses	15 (3.7)
Patients or their relatives	133 (32.9)
During your career, have you witnessed any episodes of physical violence?	
Yes	326 (80.7)
No	78 (19.3)
From your experience, who is, in general, most frequently physically harassed?	
Male	77 (19.1)
Female	105 (26.0)
Both	221 (54.9)
If you experience any form of physical violence, do you describe this/these experience/s as being very upsetting and of major importance?	
Yes, all of them	205 (50.7)
Yes, some of them	128 (31.7)
No, not all	71 (17.6)

Academic violence among participants

The prevalence of academic violence was 79%. Approximately 37.1% of the participants reported their questions/queries were intentionally not answered, 33.7% reported they were asked to carry out some personal services unrelated to patient care or educational activities, 33.2% were excluded from otherwise reasonable learning opportunities offered to others, and 27.2% were forced to refer patients without providing a reasonable cause for referral. Academic violence had occurred more frequently in the surgery (16.3%) and medicine departments (16.3%). The main source of academic violence was consultants (45.5%). During their career, 85.4% of the participants had witnessed an episode of academic violence, and they reported that both males and females were academically harassed with a similar frequency (74.0%). The majority (44.1%) of the participants reported that their experiences with academic violence were very upsetting and of major importance (Table 4).

**Table 4:** Academic violence among participants.

Variables	No. (%)
Experienced academic violence	319 (79)
Did you experience any of the following forms of academic violence?	
You were asked to carry out some personal services unrelated to patient care or educational activities	136 (33.7)
You were assigned tasks as punishment	103 (25.5)
Your questions/queries were intentionally not answered	150 (37.1)
You were excluded from otherwise reasonable learning opportunities that were offered to others	134 (33.2)
You were forced to refer patient without providing reasonable cause for referral	110 (27.2)
You were asked to take consent from very complicated cases	78 (19.3)
You were forced to do procedures that you were not mastering without supervision	98 (24.3)
You were forced to hold bleeps of senior doctor/s	93 (23.0)
You were threatened with failure or giving poor evaluations for reasons unrelated to your academic performance	93 (23.0)
Others	83 (20.5)
None of the above	85 (21.0)
in which department/s these types of academic violence have happened more frequently?	
Surgery	66 (16.3)
Medicine	66 (16.3)
Pediatrics	41 (10.1)
Ob\gyn	59 (14.6)
Emergency	49 (12.1)
ICU	17 (4.2)
Psychiatry	9 (2.2)
Other	33 (8.2)
Who was/were the main source/s of academic violence?	
Consultant	184 (45.5)
Specialist/registrars	3 (0.7)
Residents	71 (17.6)
Nurses	72 (17.8)
Patients or their relatives	20 (5.0)
During your career, have you witnessed any episodes of academic violence?	
Yes	345 (85.4)
No	59 (14.6)
From your experience, who is, in general, most frequently academically harassed?	
Male	29 (7.2)
Female	76 (18.8)
Both	299 (74.0)
If you experience any form of academic violence, do you describe this/these experience/s as being very upsetting and of major importance?	
Yes, all of them	178 (44.1)
Yes, some of them	159 (39.4)
No, not all	66 (16.3)

Sexual harassment among participants

Sexual harassment was reported by 76.5% of the participants. The most common form of sexual harassment was “received jokes or comments against your gender” (42.3%) and “faced with an offensive body language” (39.6%). Sexual harassment occurred more frequently in the surgery (36.1%) and medicine departments (26.7%). Patients or their relatives (45.3%) and consultants (40.1%) were the main sources of sexual harassment. The majority had witnessed an episode of sexual harassment during their career (81.1%) and reported that females were most frequently sexually harassed (56.9%). The majority (58.2%) reported that their experiences with sexual harassment were very upsetting and of major importance (Table 5).

**Table 5:** Sexual harassment among participants (n=404).

Variables	No. (%)
Experienced sexual harassment	309 (76.5)
Did you experience any of the following forms of sexual harassment?	
Received jokes or comments against your gender	171 (42.3)
Received compliments or comments about your body or figure	154 (38.1)
Faced with offensive body language (e.g. repeated leering, standing too close)	160 (39.6)
Was offered unwanted gifts or help to attract you	65 (16.1)
Was offered private sessions or better grades in exchange for an affair	49 (12.1)
Experience inappropriate touching of a sexual nature	112 (27.7)
Others	155 (38.4)
None of the above	95 (23.5)
In which department/s these types of sexual harassment have happened more frequently	
Surgery	146 (36.1)
Medicine	108 (26.7)
Pediatrics	38 (9.4)
Ob\gyn	39 (9.7)
Emergency	67 (16.6)
ICU	42 (10.4)
Psychiatry	47 (11.6)
Other	45 (11.1)
Who was/were the main source/s of sexual harassment?	
Consultant	162 (40.1)
Specialist/registrars	8 (2.0)
Residents	122 (30.2)
Nurses	85 (21.0)
Patients or their relatives	183 (45.3)
During your career, have you witnessed any episodes of sexual harassment?	
Yes	327 (81.1)
No	77 (18.9)
From your experience, who is, in general, most frequently sexually harassed?	
Male	27 (6.7)
Female	230 (56.9)
Both	147 (36.4)
If you experience any form of sexual harassment, do you describe this/these experience/s as being very upsetting and of major importance?	
Yes, all of them	235 (58.2)
Yes, some of them	117 (29.0)
No, not all	51 (12.6)

Reporting violence and its effects on the participants

Most participants agreed that mistreatment of hospital staff does exist (90.1%). The majority (81.4%) had experienced at least one of the mentioned types of violence, and 39.6% did not complain or report the event to the authority. The most common reasons for not reporting were “reporting would not accomplish anything” (49.4%), “reporting would become more troublesome than it was worth” (48.1%), “I was afraid that reporting would adversely affect my evaluation or my professional career in the future” (30%), “I was afraid that the reporting would not be kept confidential” (28.8%), “I did not know to whom I should report or how to complain” (27.5%) and “I was afraid of not being believed or the problem would not be dealt with fairly” (26.9%). The majority (77.5%) of the participants thought that violence is a big problem to be raised.

Most participants (77.0%) agreed that the experience that they had during their career influenced their work practice, and 69.6% of them agreed that this experience affected their view of the health care profession. About half of the participants (49.8%) said they would not advise any of their relatives to join any job related to health care (Table 6).

**Table 6:** Reporting workplace violence and its effects and influences on the participants (n=404).

Variables	No. (%)
In general, do you agree that mistreatment of hospital staff does exist	
Yes	364 (90.1)
No	40 (9.9)
In general, did you experience any of the mentioned types of violence?	
Yes	329 (81.4)
No	75 (18.6)
If yes, how often did you complain or report the event to an authority?	
None	160 (39.6)
Just once	97 (24.0)
Few times	105 (26.0)
Many times (4 or more)	42 (10.4)
If none, what was/were the reason/s for not reporting such experience	
I did not recognize the experience as harassment at the time that it happened	35 (21.9)
It was not significant to be reported to the authority	37 (23.1)
Reporting would not accomplish anything	79 (49.4)
Reporting would become more troublesome than it was worth	77 (48.1)
I dealt with the problem directly myself	39 (24.4)
I did not know to whom I should report or how to complain	44 (27.5)
I was afraid that reporting would adversely affect my evaluation or my professional career in the future	48 (30.0)
The abuser apologized to me	15 (9.4)
I was afraid of not being believed or the problem would not be dealt with fairly	43 (26.9)
I was afraid that the reporting would not be kept confidential	46 (28.8)
Others	10 (6.2)
Do you think workplace violence is a big problem to be raised	
Not a problem	20 (5.0)
Minor	71 (17.6)
Major	313 (77.5)
Did the experience that you had during your career influence your work practice?	
Yes	311 (77.0)
No	93 (23)
Did the experience affect your view of the health care profession?	
Yes	281 (69.6)
No	123 (30.4)
If yes, have you advised any of your relatives not to join any job related to healthcare?	
Yes	201 (49.8)
No	203 (50.2)

The association between the demographics of the participants with workplace violence

In this study, no significant association was found between the socio-demographical and work profile of the participants and the experience of violence.

## Discussion

In this study, 81.4% of the participants experienced verbal, physical, academic, or sexual violence. This prevalence is higher than that found in previous similar studies conducted in Arar City and Abha City. In those studies, the prevalence of workplace violence among healthcare workers was 57.5% and 48.5% in Abha City [15] and Arar City [16], respectively. In other local studies that focused on a particular setting or occupation, the prevalence of workplace violence ranged between 90.7% in emergency departments in Tabuk [12] to 27.7% in primary care centers in Al-Hassa [14]. Internationally, the prevalence of workplace violence against healthcare workers was between 48.1% in Europe and 70.9% in Australia and New Zealand [8]. The high rates of violence against healthcare providers could be due to the nature of their jobs, which exposes them to work in close contact with people in times where they are in unstable conditions like confusion, aggression, or drug or alcohol intoxication [18,19]. Moreover, high levels of tension, stress, and anxiety during the time of admissions or hospital visits may contribute to this prevalence [20]. High expectations, increased demands, and long waiting times for the patients or their families were also found to cause violence [21]. These factors can explain the difference in workplace violence in different studies. For example, the difference between the prevalence found in this study (81.4%) compared to the studies that were conducted in Abha City (57.5%) [15] and Arar City (48.5%) [16] could be attributed to the fact that Abha and Arar are peripheral cities in Saudi Arabia and they have a much smaller population compared to Riyadh, which could lead to their health centers being less overwhelmed with patients resulting in less waiting times, frustration, and violence from the patients.

Verbal violence was found to be the most common type of violence in this study with 79.5% of the participants reporting that they encountered verbal violence compared to 79% who encountered academic violence, 76.5% who encountered sexual harassment, and 67.6% who encountered physical violence. Verbal violence was also the most common type in Arar City, with 83% of those who were subjected to violence in that study being verbally abused [15]. Similarly, 55.9% of violence victims were verbally abused in Abha City [16]. Verbal violence was also the most common type of workplace violence among healthcare workers in other Saudi studies [9-14], and it was found to be the most common type of violence in an international literature review that included studies from Asia, Europe, America, Africa, and Australia [8]. In this study, patients or their relatives were the main sources of violence of all violence categories (verbal, physical, and sexual) except academic violence in which consultants were the main source. This finding is consistent with previous local studies conducted in different settings [9-16], and international studies in Europe [22] and Canada [23], which arguably indicates the need for awareness programs targeting the general population regarding the negative impact of violence against healthcare providers on the quality of health care.

Participants in our study reported that they experienced verbal and physical violence mostly in the emergency department (40.3% and 18.1%, respectively), while sexual harassment was frequently experienced in the surgery department (36.1%), and academic violence was experienced in the medicine and surgery departments (16.3%). Emergency departments were described to have high rates of violence against health care workers [24], perhaps due to overcrowding, long waiting times, and lack of privacy, which cause frustration and anger among patients [24]. Also, emergency departments have high rates of patients who come intoxicated [24].

Both males and females were perceived to being equally harassed in all categories of violence in our study, except sexual harassment in which females were perceived to be more frequently harassed by participants who have witnessed incidents of harassment. However, no statistically significant associations were found between gender and any type of violence. Previous studies were not consistent in finding an association between gender and workplace violence [25-27]. However, female healthcare providers were found to have a higher risk of sexual harassment than their male counterparts [28]. The absence of statistical associations between violence and the variables in this study indicates that all healthcare workers were subjected to violence regardless of their gender, age, specialty, nationality, years of experience, or job title. Accordingly, any effort to address violence among health care workers should arguably target everybody equally.

In this study, 39.6% of the participants did not report the violence that they were subjected to because they thought “reporting would not accomplish anything” (49.4%), or “reporting would become more troublesome than it was worth” (48.1%). This could be due to the belief that violence in healthcare settings is “part of the job” or that the patients are not in full control of their actions, which are common reasons for not reporting [29]. Another common reason for not reporting violence in this study was “I was afraid that reporting would adversely affect my evaluation or my professional career in the future” (30%). This could be attributed to the fear of retaliation, which was described as a reason for not reporting workplace violence in previous studies [30]. Similarly, healthcare workers in previous Saudi studies did not report the violent incidents that they encountered because they thought reporting was useless [15] or not an effective reaction [13]; while others did not report because they were afraid of the consequences of reporting [15] or had previous negative experiences were reporting did not accomplish anything [12]. This shows that there is a substantial need for hospitals to establish clear guidelines regarding workplace violence and outline how such violence would be handled by the institution. About one-third of the participants in this study reported that they “did not know to whom they should report or how to complain” (27.5%), which obligates hospitals and health care settings to educate their employees regarding the reporting of workplace violence. We highly recommend that detailed information about workplace violence and how to report it should be incorporated into the orientation programs that are usually given to new employees at healthcare facilities across Saudi Arabia.

The majority (77%) of the participants reported that their experience with workplace violence influenced their work practice. Previous studies have established an association between workplace violence and major occupational impacts like increased absence [6], wanting to quit [7], and less productivity [4]. According to a review published in 2014, up to 60% of those who were subjected to violence in the workplace thought about quitting their profession [3]. The duration of sick leaves taken by people who were subjected to violence was from 1 to 7 days [3]. We recommend that mental health services should be offered to healthcare workers who report incidents of workplace violence.

This study has some limitations. The fact that it is based on a survey, which subjects the results to self-reporting bias. Also, this study was conducted in only one medical city, which is considered another limitation.

## Conclusions

High rates of workplace violence were found in this study. Approximately one-third of violence victims did not report the incidents thinking that “reporting would not accomplish anything”, and another one-third of the victims did not know how to report, which requires healthcare facilities to establish clear guidelines and penalties for workplace violence. There is arguably an urgent need to develop programs that can reduce workplace violence and facilitate reporting it in hospitals. The majority of the participants reported that their experience with workplace violence influenced their work practice, which suggests that mental health services should be implemented for those who encountered workplace violence. Moreover, community-based awareness programs regarding the negative impacts of violence against healthcare workers on the quality of care are needed due to the high prevalence of violence by patients or their relatives.
